# Liver fibrosis secondary to bile duct injury: correlation of Smad7 with TGF-β and extracellular matrix proteins

**DOI:** 10.1186/1471-230X-9-81

**Published:** 2009-10-31

**Authors:** María del Pilar Alatorre-Carranza, Alejandra Miranda-Díaz, Irinea Yañez-Sánchez, Oscar Pizano-Martínez, José M Hermosillo-Sandoval, Mónica Vázquez-Del Mercado, Sebastián Hernández-Hoyos, Ricardo Martínez-Abundis, Mary Fafutis-Morris, Jorge Segura-Ortega, Vidal Delgado-Rizo

**Affiliations:** 1 Departamento de Fisiología, Centro Universitario de Ciencias de la Salud, Universidad de Guadalajara; Sierra Mojada 950, Col. Independencia, Guadalajara, Jalisco 44340 México; 2 Hospital de Especialidades Centro Médico Nacional de Occidente, IMSS; Belisario Domínguez 1000, Col. Independencia, Guadalajara, Jalisco 44340 México; 3 Instituto de Investigación en Reumatología y Sistema Músculo Esquelético (IIRSME), Universidad de Guadalajara; Sierra Mojada 950, Col. Independencia, Guadalajara, Jalisco 44340 México; 4 Servicio de Gastroenterología Hospital Civil de Guadalajara, Universidad de Guadalajara, Hospital 278, Col. El Retiro, Guadalajara, Jalisco 44280 México; 5 Departamento de Reumatología, Hospital Civil de Guadalajara "Dr. Juan I. Menchaca", Universidad de Guadalajara, Salvador Quevedo y Zubieta 750, Col. Independencia, Guadalajara, Jalisco 44340 México

## Abstract

**Background:**

Liver fibrosis is the result of continuous liver injury stemming from different etiological factors. Bile duct injury induces an altered expression of TGF-β, which has an important role in liver fibrosis because this cytokine induces the expression of target genes such as collagens, PAI-1, TIMPs, and others that lead to extracellular matrix deposition. Smad7 is the principal inhibitor that regulates the target gene transcription of the TGF-β signaling. The aim of the study was to determine whether Smad7 mRNA expression correlates with the gene expression of *TGF-β, Col I*, *Col III*, *Col IV*, or *PAI-1 *in liver fibrosis secondary to bile duct injury (BDI).

**Results:**

Serum TGF-β concentration was higher in BDI patients (39 296 pg/ml) than in liver donors (9008 pg/ml). Morphometric analysis of liver sections showed 41.85% of tissue contained fibrotic deposits in BDI patients. mRNA expression of Smad7, Col I, and PAI-1 was also significantly higher (*P *< 0.05) in patients with BDI than in controls. Smad7 mRNA expression correlated significantly with TGF-β concentration, Col I and Col III expression, and the amount of fibrosis.

**Conclusion:**

We found augmented serum concentration of TGF-β and an increase in the percentage of fibrotic tissue in the liver of BDI patients. Contrary to expected results, the 6-fold increase in *Smad7 *expression did not inhibit the expression of *TGF-β, collagens*, and *PAI-1*. We also observed greater expression of Col I and Col III mRNA in BDI patients and significant correlations between their expression and TGF-β concentration and Smad7 mRNA expression.

## Background

Liver fibrosis is caused mainly by alcohol consumption and viral hepatitis, and it is an important health problem throughout the world. Bile duct injury (BDI) is also an important etiological factor because it can cause secondary biliary cirrhosis and long-term disability, and it increases the risk of death threefold[1,2]. BDI can occur as a complication of cholecystectomy, an elective surgery for cholelithiasis. The introduction of laparoscopy has increased the BDI incidence to 0.3-1.0%, which is higher than that associated with open cholecystectomy (0.1-0.3%) [3-5].

BDI causes obstruction of bile ducts, which results in cholestasis. BDI increases the concentrations of serum aminotransferases, bilirubin, and alkaline phosphatase (AP). As the obstruction proceeds, the accumulation of biliary salts in the canalicular membrane produces dilatation of bile ducts, which may rupture and form bile deposits. Mononuclear cells infiltrate into the portal tracts and bile ducts proliferate, leading to degeneration of hepatocytes and periportal fibrosis deposition[3,6-8].

Transforming growth factor-β (TGF-β) is a multifunctional cytokine involved in the regulation of cell proliferation, differentiation, extracellular matrix (ECM) production, wound healing, and tissue repair. TGF-β plays an important role in liver fibrogenesis because it triggers the overexpression and deposition in the ECM of molecules such as PAI-1, TIMP-1, Col I, Col III, Col IV, tenascin, fibronectin, and Smad7 [9-14].

Smad7 is the principal inhibitor that regulates the target gene transcription of the TGF-β signaling[15,16]. Smad7 exerts its inhibitory effect by its association with activated TGF-β I receptor and inhibition of Smad2/3 phosphorylation.

Smad7 has been used in experimental models to block TGF-β effects in different disorders [17-19]. Dooley et al[20] and Tahashi et al[16] demonstrated that Smad7 expression was low in experimental liver fibrosis, and this event allows liver scar formation. Some investigators have used gene therapy[16,20,21] with an adenoviral vector containing Smad7 complementary DNA (cDNA) and a Chinese herbal medicine[22], to treat fibrosis and cirrhosis in different experimental models. However, there are no reports on Smad7 in human liver fibrosis. In this context, the aim of our study was to determine whether Smad7 mRNA expression correlates with the expression of *TGF-β*, *Col I*, *Col III*, *Col IV*, *and PAI-1 *genes in BDI patients and to compare these relationships with controls (liver donors).

## Methods

### Patients and samples

Eighteen patients were included in this study. Fourteen patients had extrahepatic BDI caused by open or laparoscopic cholecystectomy that had been reconstructed surgically. These patients were recruited from Centro Médico General de Occidente, and 4 liver donors with normal liver histology, recruited from the Hospital Civil de Guadalajara, were included as a control group. The serum concentrations of alanine aminotransferase (ALT), aspartate aminotransferase (AST), direct bilirubin (DB), and AP were measured before the reconstruction surgery. Serum was obtained from a peripheral blood (PB) sample without anticoagulant from each patient and control. Liver biopsy specimens were obtained during bile duct reconstruction surgery. None of the patients or controls had a clinical history of previous liver disease or alcohol or drug abuse. The study protocol was in accordance to the ethical guidelines of the World Medical Association Declaration of Helsinki (adopted by the 52^nd ^WMA General Assembly, Edinburgh, Scotland, October 2000) and was approved by the ethical committee of the institutions. Informed written consent was obtained from each patient and control.

### Histological analysis

The liver specimens were fixed immediately in 4% paraformaldehyde solution, buffered to pH 7.4, and embedded in paraffin. Five-micrometer sections were cut and stained with Masson's trichrome. The proportion of each field displaying fibrosis was analyzed using computer image analysis software (Image Pro Plus 4.0). Briefly, regions of the section with the optical density characteristics of fibrosis where counted and analyzed as a percentage of the total tissue. The total collagen surface density was measured in 20 random fields at a magnification of 20× and is expressed as the percentage of total section area.

### ELISA for TGF-β

An ELISA kit (R&D Systems) was used to measure the serum concentration of TGF-β following the manufacturer's protocol. The samples were activated by adding 200 μl in a tube with 200 μl of 2.5 M acetic acid, mixed, incubated for 10 min at room temperature, and inactivated with 200 μl of 2.7 M NaOH in 1 M HEPES. One hundred microliters of the activated sample, negative control, and the standards were added to the plate in triplicate. The absorbance was measured at 450 nm.

### RNA extraction

Total RNA was extracted from a section of each liver biopsy using Trizol (Invitrogen™) according to the manufacturer's instructions. The tissue was homogenized with 500 μl of Trizol. Chloroform (Sigma) was added and the sample was centrifuged at 10,000 rpm at 4°C. The total RNA was precipitated with isopropanol (Sigma). The RNA pellet was washed with 75% ethanol and dissolved in diethylene pyrocarbonate (DEPC) water. The final concentration and quality of RNA were determined by spectrophotometry, and the absorbance was measured at 260 and 280 nm.

### Reverse transcription and real-time Polymerase chain reaction (PCR)

Total RNA was reverse transcribed to cDNA using SuperScript III (Invitrogen™) enzyme. Two hundred nanograms of total RNA, 1 μl of 10 mM dNTP mix, 1 μl of (0.125 μg/μl) random primers, and DEPC water up to 13 μl were incubated at 70°C for 5 min. Four microliters of 5× first-strand buffer, 1 μl of 0.1 M DTT, 1 μl of RNaseOUT, and 1 μl of SuperScript III were added, and the samples were incubated at 25°C for 5 min (annealing), at 50°C for 50 min (cDNA synthesis), and at 72°C for 15 min (enzyme denaturation).

Real-time PCR was performed with an ABI Prism 7500 Sequence Detection System (Applied Biosystems). Two microliters of cDNA was used in each PCR reaction. The housekeeping gene 18S rRNA (Applied Biosystems) was used for normalization and water was used as a no-template control. The primers and probes (Applied Biosystems) for the PCR reaction were those for the target genes *TGF-β*, *Col I*, *Col III*, *Col IV*, *PAI-1*, and *Smad7 *(Table 1).

**Table 1:** Primers and probes of the genes studied

Gene	GenBank (Accession number)	Applied Biosystems ID	Dye
18S rRNA	--	FG 18S RNA	VIC
*TGF-β*	NM000660	HS00171257_m1	FAM
*Col I*	NM000089	Hs00164099_m1	FAM
*Col III*	NM000090	Hs00164103_m1	FAM
*Col IV*	NM_001845	Hs00266237_m1	FAM
*PAI-1*	NM_000602	Hs00167155_m1	FAM
*Smad7*	NM005904	Hs00178696_m1	FAM

Each sample was analyzed in triplicate. Two microliters of each cDNA sample was mixed with 7 μl of DEPC water, 10 μl of Universal Master Mix (Applied Biosystems), and 1 μl of primer and probe (Applied Biosystems) of either the housekeeping or the target gene. Twenty microliters of this mixture was added to each well for each sample.

The relative quantification was calculated using the ΔΔC_T _method. The mean C_T _of triplicate samples was used to calculate the ΔC_T _as the difference in C_T _between the target and reference gene. ΔC_T _for each sample minus the experimental reference ΔC_T _control was expressed as the ΔΔC_T_. The relative quantification is expressed as the fold expression of the target gene compared with the reference control expression according to the formula 2^-ΔΔCT ^and expressed in relative expression units (REU).

### Statistics

The results are expressed as mean ± SD. Student's *t *test was used to analyze differences between groups, and the Spearman (r_s_) correlation was used to analyze relationships between variables. *P *values < 0.05 were considered significant.

## Results

### Biochemical studies

The 14 BDI patients (10 women and 4 men) had a mean age of 43.9 years (range, 17-65 years), and the 4 controls (liver donors) had a mean age of 37.2 years (range, 21-61 years). The BDI patients had higher concentrations of the aminotransferases, DB, and AP (Table 2).

**Table 2:** Clinical and biochemical features of control and bile duct injury (BDI) patients groups

Group	N	Bismuth (lesion type)	Time post-lesion (days)	ALT (0-45 UI/l)	AST (0-41 UI/l)	DB (0.1-0.4 mg/dl)	AP (30-115 UI/l)
Control	1	0^†^	0	16	18	0.36	21
	2	0^†^	0	5	6	0.29	68
	3	0^†^	0	26	23	0.43	73
	4	0^†^	0	12	19	0.12	49
BDI	1	2	49	265	110	3.70	380
patients	2	1	2190	365	479	3.70	740
	3	3	49	133	127	0.20	391
	4	3	489	54	58	5.10	284
	5	3	43	132	98	5.10	114
	6	3	56	197	79	0.90	378
	7	3	NA	273	22	2.80	899
	8	2	145	133	58	2.90	860
	9	3	65	164	168	2.00	357
	10	3	70	338	40	.01	489
	11	3	42	285	85	6.20	806
	12	3	1	106	95	23	320
	13	3	1825	140	NA	0.90	306
	14	3	56	222	117	NA	296

^† ^= without lesion

NA = not available

### Bismuth classification

According to the Bismuth classification method for iatrogenic BDI[23], 11 patients were classified with lesion type 3, 2 with lesion type 2, and 1 with lesion type 1 (Table 2). The lesion type did not correlate with the mRNA expression, with the extent of fibrosis, or serum TGF-β concentration. Further experiments, with a larger patient group, will be necessary to prove if the type or severity of BDI does or does not influence the liver fibrosis process.

### Liver fibrosis

**Figure 1:**
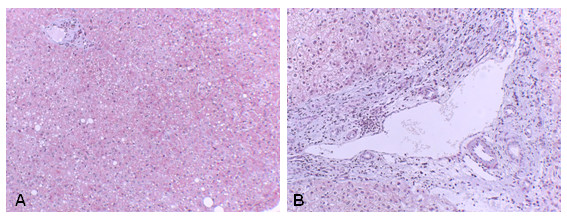
**Liver fibrosis**. The liver section stained with Masson's trichrome from a control (A), compared with a BDI patient (B) showed significant deposition of ECM proteins (blue stain) in B.

**Figure 2:**
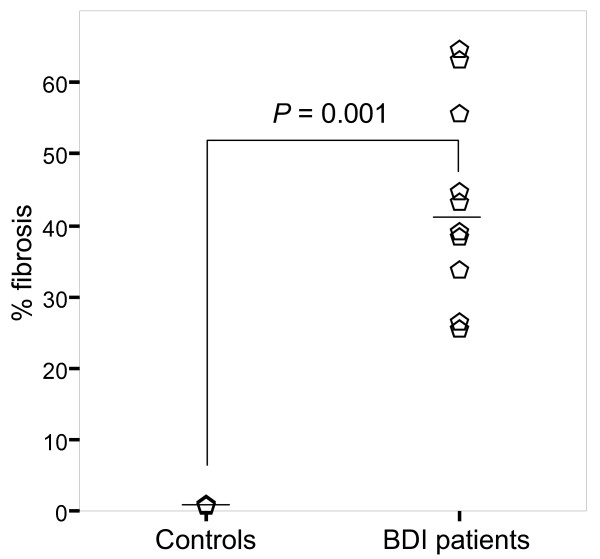
**Percent optical density of collagen staining**. Morphometric analysis demonstrates more fibrosis in a liver section from BDI patients, who had an average of 41.85% fibrosis in liver tissue, which differed significantly from the percentage in the control group.

Liver biopsies stained with Masson's trichrome stain showed significant collagen levels in BDI patients (Figure 1B) we correlated collagen levels with increases in fibrosis. Less of the ECM was stained in the livers of the controls (Figure 1A). The morphometric analysis was correlated with an average of 41.85% fibrosis in the BDI group compared with 0.83% fibrosis in the control group (*P *= 0.001) (Figure 2). The increase in collagen staining did not correlate with the lesion type in the BDI patient group, probably because of the variety of types in these patients. In the morphological examination of liver biopsies from patients, we observed cholestasis, fibrosis around portal triad, proliferation of bile ducts and periportal inflammatory infiltrate.

### Serum TGF-β concentration

**Figure 3:**
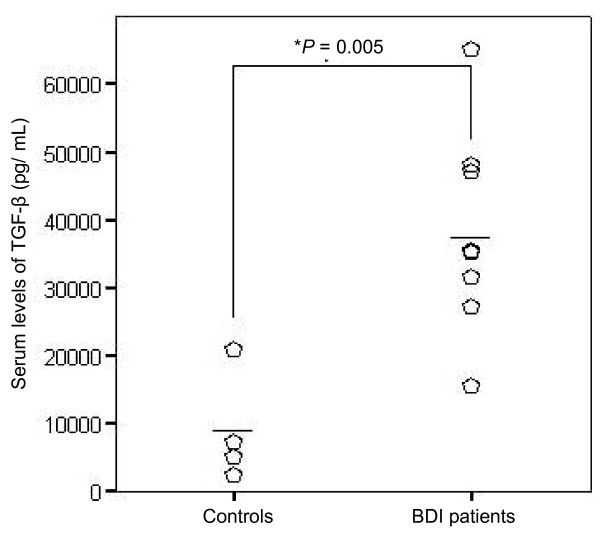
**Serum concentrations of TGF-β in BDI patients and controls**. TGF-β concentrations were higher in BDI patients, who had a mean concentration of 39 296.36 pg/ml (*P *= 0.005 compared with controls).

TGF-β concentration was measured in the PB of only 9 of the 14 BDI patients because it was not possible to obtain blood samples from all patients. Serum TGF-β concentration was significantly higher in the patients than in the controls (Figure 3). The mean concentrations were 39 296 pg/ml (range, 15 565-65 194 pg/ml) in patients and 9008 pg/ml (range, 2622-20 880 pg/ml) in controls (*P *= 0.005).

### *TGF-β*, *Smad7*, *Col I*, *Col III*, *Col IV*, and *PAI-1 *gene expression

**Figure 4:**
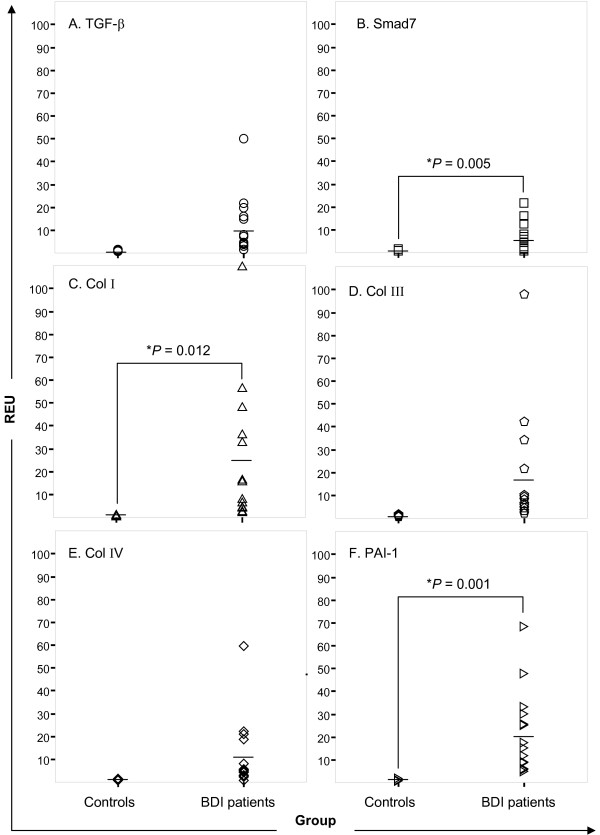
**mRNA expression of TGF-β and gene targets by real-time PCR**. TGF-β (A), Smad7 (B), Col II (C), Col III, (D), Col IV (E), and PAI-1 (F) mRNA levels were higher in BDI patients than in controls, although the differences were significant only for Smad7, Col I, and PAI-1 expression.

To evaluate the differential gene expression, we quantified the level of TGF-β mRNA expression and its target genes, *Smad7*, *Col I*, *Col III*, *Col IV*, and *PAI-1 *in both groups. The mean TGF-β mRNA level was 11-fold higher in BDI patients (11.59 REU) than in the controls (liver donors) (*P *= 0.138) (Figure 4A). Smad7 mRNA expression showed a similar pattern: 6.69 REU in patients and 1.12 REU in controls (*P *= 0.005) (Figure 4B).

Collagen mRNA expression was higher in the BDI group. The mean level of Col I mRNA was 26.86 REU in patients and 1.35 REU in controls (P = 0.012) (Figure 4C). The BDI patients showed a homogeneous pattern of Col III, mRNA expression (mean 18.84 REU) (Figure 4D). Col IV (mean 11.77 REU; Figure 4E) and PAI-1 (mean 22.18 REU, *P *= 0.001; Figure 4F) were also overexpressed in BDI patients.

The Spearman (r_s_) correlation showed that the Smad7 mRNA level correlated positively with the level of TGF-β, Col I, and Col III, mRNA (Table 3). As expected, the mRNA expression of all analyzed genes, except for Col *IV*, correlated significantly with the increase in serum TGF-β concentration (data not shown), probably because of the direct influence of TGF-β signaling on the expression of ECM proteins.

**Table 3:** Correlation of Smad7 mRNA expression with the expression of TGF-β, Col I, Col III, Col IV, and PAI-1

Variable	Spearman correlation (r_s_)	*P *value
TGF-β	0.815	*0.000
Col I	0.753	*0.003
Col III	0.886	*0.000
Col IV	0.209	0.474
PAI-1	0.468	0.091

* = *P *< 0.05

## Discussion

BDI causes biliary acid flow obstruction, increases bile deposition in hepatic tissue, and causes hepatocyte degeneration, cell death, and release of liver enzymes such as aminotransferases and AP. Additionally, inflammatory cells migrate to the injured tissue, which contributes to secondary biliary cirrhosis[7,8].

TGF-β is a pleiotropic molecule involved in ECM proteins expression in fibrogenesis[24,25]. There is some controversy about whether the serum TGF-β concentration can be correlated with hepatic injury in different liver diseases[26,27]. Despite this controversy, we found that BDI patients have increased serum concentrations of this protein, perhaps reflecting the overexpression of this cytokine in the liver. mRNA TGF-β expression also increases in rat liver cholestasis[20,28].

Dooley et al[20] demonstrated that *Smad7 *(overexpressed through an adenovirus containing Smad7 cDNA) inhibits TGF-β signal transduction and influences the extent of fibrosis when used as preventive therapy in a bile duct ligation (BDL) model of fibrosis. These results suggest that Smad7 is a potential therapeutic tool for liver fibrosis and cirrhosis. The aim of our study was to determine whether *Smad7 *expression correlates with the gene expression of TGF-β, Col I, Col III, Col IV, and PAI-1 in patients with liver fibrosis caused by BDI.

Tahashi et al[16] showed that Smad7 protein has a critical role in acute and chronic liver injury and that the amount of this protein increases in acute and decreases in chronic liver injury, along with an associated decrease in collagen expression. Our results showing increased Smad7 mRNA expression along with the collagens I, III and IV seem to contradict those of Tahashi et al. However, they used a CCl_4 _model that produces damage similar to that caused by alcohol in humans and not a BDL model.

We found that *TGF-β *mRNA expression was 11-fold higher in BDI patients than in liver donors. This contrasts with a 6-fold higher *Smad7 *expression level in BDI patients. An increase in the expression of *Smad7 *must correlate with a reduction of the expression of fibrogenic molecules because of the direct negative feedback in TGF-β signaling. Although we found higher Smad7 mRNA expression in patients, expression of the mRNA for collagens and PAI-1 also increased, demonstrating that *Smad7 *expression is not enough to inhibit TGF-β signaling. These results are similar to those reported recently by Seyhan et al[29] who found continuous *Smad7 *augmentation along with liver fibrosis cholestasis induced in an experimental rat model was insufficient to blunt fibrogenesis. This result may reflect the observation that the transcriptional regulation of ECM proteins can be directed through another TGF-β dependent pathway[30], such as connective tissue growth factor (CTGF), a growth factor that is recognized as a fibrogenic molecule in the liver[31,32]. Smad7 expression is regulated by two mechanisms: transcription and protein degradation[33]. The Smad7 transcriptional regulation is mediated by the Ski protein, which inhibits the Smad7 promoter, and the posttransductional regulation is mediated through the effects of Smurf and Jab1 proteins, which promote Smad7 degradation[34,35].

We found a strong positive correlation of Smad7 mRNA expression with TGF-β, Col I and Col III mRNA expression. The mRNA expression levels were 6.69, 11.59, 26.86, and 18.84 REU, respectively. We also observed a significant amount of fibrotic tissue deposition in the liver biopsies of BDI patients. In the literature, there is one report on increased Smad7 levels in tissue biopsies[36]. In this study, we were limited to analyzing only mRNA expression, and the expression of Smad7 and of ECM proteins need to be measured in BDI patients.

We emphasize that most of the information about the fibrotic process and the molecules involved had been obtained from experimental models (animals and cell cultures). This is the first work in humans to focus on the mRNA expression of ECM proteins and their relationship to Smad7.

The clinical parameters of biliary obstruction evaluated in the patients (AST, ALT, DB, and ALP concentrations) did not correlate with the gene expression levels, serum TGF-β concentration, or fibrosis percentage. We found no correlation between the lesion type and fibrosis percentage. Type 3 lesion was the most frequent lesion in the patients (11 from 14), but this type was not related to the mRNA expression or liver fibrosis.

Additionally, gene polymorphisms of TGF-β (non coding -509 C/T and coding codon 10, codon 25, codon 263) have been associated with modified serum levels of this cytokine in different diseases [37-39]. We showed increased serum levels of TGF-β in patients with BDI. In this study, we did not evaluate any of the TGF-β polymorphisms. It will be important to plan further studies to determine if serum TGF-β levels are more affected by the polymorphisms rather than the presence of bile duct injury.

A recent report demonstrated the predominance of a single nucleotide polymorphism at codon 25 in cirrhotic, but not healthy subjects of Mexican origin[40]. It will also be interesting to determine if there exists a strong relationship between Smad7 and TGF-β dependent genes such as collagens, PAI-1, TIMPs, and others that lead to extracellular matrix deposition in patients with different polymorphisms for TGF-β.

## Conclusion

We found augmented serum concentration of TGF-β and an increase in the percentage of fibrotic tissue in the liver of BDI patients. Contrary to expected results, the 6-fold increase in *Smad7 *expression did not inhibit the expression of *TGF-β, collagens*, and *PAI-1*. We also observed greater expression of Col I and Col III mRNA in BDI patients and significant correlations between their expression and TGF-β concentration and Smad7 mRNA expression.

## Competing interests

The authors declare that they have no competing interests.

## Authors' contributions

MPAC performed the experimental work and data analysis, searched the scientific literature, and drafted and edited the manuscript. AMD participated in the design of the project and gave funding support. IYS and OPM participated in the collection of samples. JMHS, SHH, and RAM were responsible for obtaining clinical data from the patients. MVM contributed substantially to the writing of the manuscript, MFM and JSO contributed to the conceptualization of the study, and VDR conceived and designed the study. All authors read and approved the final manuscript.

## Pre-publication history

The pre-publication history for this paper can be accessed here:

http://www.biomedcentral.com/1471-230X/9/81/prepub
